# Obacunone Protects Against Ulcerative Colitis in Mice by Modulating Gut Microbiota, Attenuating TLR4/NF-κB Signaling Cascades, and Improving Disrupted Epithelial Barriers

**DOI:** 10.3389/fmicb.2020.00497

**Published:** 2020-03-31

**Authors:** Xiaoping Luo, Bei Yue, Zhilun Yu, Yijing Ren, Jing Zhang, Junyu Ren, Zhengtao Wang, Wei Dou

**Affiliations:** Shanghai Key Laboratory of Formulated Chinese Medicines, Institute of Chinese Materia Medica, Shanghai University of Traditional Chinese Medicine, Shanghai, China

**Keywords:** UC, gut microbiota, TLR4/NF-κB, epithelial barrier, obacunone

## Abstract

Obacunone, a natural limonoid compound abundantly distributed in citrus fruits, possesses various biological properties, such as antitumor, antioxidant, and antiviral activities. Recent studies suggested an anti-inflammatory activity of obacunone *in vitro*, but its efficacy on intestinal inflammation remains unknown. This study was designed to evaluate the effects and mechanisms of obacunone in ameliorating intestinal inflammation in a mouse model of ulcerative colitis (UC). We found that obacunone efficiently alleviated the severity of dextran sulfate sodium (DSS)-induced mouse UC by modulating the abnormal composition of the gut microbiota and attenuating the excessive activation of toll-like receptor 4 (TLR4)/nuclear factor-kappa B (NF-κB) signaling. The intestinal epithelial barrier was disrupted in DSS colitis mice, which was associated with activation of inflammatory signaling cascades. However, obacunone promoted the expression of tight junction proteins (TJP1 and occludin) and repressed the activation of inflammatory signaling cascades. In summary, our findings demonstrated that obacunone attenuated the symptoms of experimental UC in mice through modulation of the gut microbiota, attenuation of TLR4/NF-κB signaling cascades, and restoration of intestinal epithelial barrier integrity.

## Introduction

Inflammatory bowel diseases (IBDs), consisting mainly of Crohn’s disease and ulcerative colitis (UC), are common, chronic, and relapsing inflammatory disorders of the digestive tract (Kaplan, 2015). The clinical features of UC include recurrent, chronic, and persistent inflammation in the gastrointestinal tract. Furthermore, the symptoms of UC frequently include diarrhea, abdominal pain, weight loss, and malnutrition, which seriously affect the quality of life of UC patients (Rosen et al., 2015). The possible etiology of UC is complex and multifactorial including genetic, immune, microbiological, and environmental factors, each of which may lead to the occurrence of UC (Bernstein, 2017). Although the exact cause of UC remains unclear, accumulating evidence has indicated that interaction between mucosal immunity and gut microbiota plays a key role in the pathogenesis of UC (Yue et al., 2019). Additionally, clinical studies have indicated that the composition of three major phyla of bacteria present in the gut microbiota of UC patients is disturbed, with a decrease in the proportion of *Firmicutes* and *Bacteroidetes* and an increase in that of *Proteobacteria* (Matsuoka and Kanai, 2015; Mirsepasi-Lauridsen et al., 2019). Administration of antibacterial agents and probiotics in UC patients has suggested that the gut microbiota does play a role in the onset of UC (Sokol, 2014).

Tight junction proteins (TJPs), localized at the interface between epithelial cells, are responsible for maintaining the integrity and function of the intestinal mucosal barrier (Bhat et al., 2018). Disruption of mucosal barrier in UC patients can trigger bacterial and endotoxin translocation, resulting in the loss of innate immunity, as well as aberrant activation of acquired immunity (Wang et al., 2019). Bacterial or endotoxin invasion is primarily recognized by toll-like receptor 4 (TLR4), a typical member of the pattern recognition receptors family. TLR4 recognizes bacterial components, such as lipopolysaccharide (LPS), which initiates a signaling cascade that results in the activation of nuclear factor-kappa B (NF-κB); this induces a series of pro-inflammatory immune responses by the host in an attempt to destroy the invading pathogens (Stephens and von der Weid, 2019; Yue et al., 2019).

There are various classical therapeutic approaches for UC, including anti-inflammatory, immunosuppressive, and biological therapies. However, a considerable number of UC patients have not benefited from these approaches, owing to insensitivity to these treatments or the presence of significant therapy-associated adverse effects (Neurath, 2017). Consequently, novel therapeutic options are constantly being developed, while traditional treatment approaches have attracted increasing attention in recent years. Obacunone is a triterpenoid limonoid compound isolated primarily from citrus fruits and plants of the *Rutaceae* family, such as *Phellodendron chinense* and *Tetradium ruticarpum* (Gao et al., 2018). Obacunone is found to display various pharmacological activities, including antioxidative, anti-inflammatory, antitumor, and hypoglycemic effects (Murthy et al., 2015). However, the effects of obacunone on UC and the underlying mechanisms have not been reported to date. In this study, we presented evidence that obacunone could alleviate UC-associated symptoms in mice, and this effect was mediated through modulation of the gut microbiota, attenuation of TLR4/NF-κB signaling cascades, and restoration of intestinal epithelial barrier integrity.

## Materials and Methods

### Chemicals and Reagents

Obacunone (C_26_H_30_O_7_, MW: 454.516, CAS: 751-03-1, HPLC purity ≥ 98%) was obtained from Meilun Biological Technology Co., Ltd. (Dalian, China). RAW264.7 mouse macrophage cells and NCM460 human colonic epithelial cells were obtained from the American Type Culture Collection (ATCC, Manassas, VA, United States). Dulbecco’s modified Eagle’s medium (DMEM), Roswell Park Memorial Institute (RPMI)-1640, and fetal bovine serum (FBS) were purchased from Gibco BRL (Grand Island, NY, United States). DEPC water, LPS, formalin, paraformaldehyde, and ethanol were from Sigma-Aldrich. Dimethyl sulfoxide (DMSO) and Tween 20 were obtained from Sangon Biotech Company (Shanghai, China). Antibodies against p-p65 (#SC-33039), p-IκBα (#SC-8404), and actin (#SC-47778) were from Santa Cruz Biotechnology (CA, United States). The anti-TLR4 (ab13556) and inducible nitric oxide (iNOS, ab129372) antibody were purchased from Abcam (Cambridge, MA, United States), while the anti-ZO-1 (A11417) and anti-occludin (A2601) antibodies were purchased from ABclonal Technology (Wuhan, China). All the other antibodies were from Cell Signaling Technology (Danvers, MA, United States), as follows: COX-2 (#12282P), IFN-γ (#3159), TNF-α (#3707S), and MyD88 (#42835). All reagents for quantitative polymerase chain reaction (qPCR) and the Reverse Transcriptase Kit were from Takara Biotechnology (Shiga, Japan). The Cell Counting Kit 8 (CCK-8) was from Meilun Biological Technology Co., Ltd. All other reagents were obtained from Thermo Fisher Scientific (Waltham, MA, United States).

### Animals and DSS-Induced Colitis

Healthy C57BL/6 mice (8 weeks of age, 20–22 g) were purchased from the Shanghai Laboratory Animal Center. All mice were maintained in specific pathogen-free facility and kept under controlled conditions at a humidity of 60–70% and stationary temperature of 23–25°C with a 12 h light/dark cycle and with access to autoclaved food and drinking water. This study was carried out in accordance with the principles of the declaration recommendations of the Animal Experimentation Ethics Committee at Shanghai University of Traditional Chinese Medicine (Animal license key: SYXK2014-0008).

All mice were randomly divided into the following six groups (*n* = 10 mice per group): vehicle control group, obacunone-only group (100 mg/kg), dextran sulfate sodium (DSS) group, and three obacunone-treated groups (low dosage, middle dose, and high dose, respectively). The dosages of obacunone were 25, 50, and 100 mg/kg/day per body weight, respectively. Acute experimental colitis was induced in mice by administration of 3.5% DSS (MW: 36,000–50,000 Da, MP Biomedicals, Irvine, CA, United States) for 7 days as previously described (Yue et al., 2018). Obacunone was dissolved in 0.5% methylcellulose and administered by oral gavage once a day, starting from 2 days before DSS treatment and continuing to the end of the experiment. Body weight and bloody diarrhea were recorded daily. Mice were euthanized under anesthesia after the last oral gavage. The entire colon was removed and the total length was measured. After that, the distal colon tissues were collected for hematoxylin–eosin (H&E) staining. And the images were taken by the Olympus DP20 optical digital microscope camera (Tokyo, Japan). Histological injury was assessed by a combined score of inflammatory cell infiltration (score 0–3) and epithelial damage (score 0–3) using a double-blind method as described previously (Luo et al., 2017).

### Cell Culture and Cell Viability Assay

Murine peritoneal macrophage RAW264.7 cells were cultured in DMEM supplemented with 10% FBS under 5% CO_2_ at 37°C. Cells were treated with different concentrations of obacunone (0–100 μM) for 24 h. NCM460 human colonic epithelial cells were cultured in RPMI-1640 supplemented with 10% FBS under 5% CO_2_ at 37°C. NCM460 cells were treated with different concentrations of obacunone (0–100 μM) for 2 h followed by coincubation with tumor necrosis factor (TNF-α) (20 ng/mL) for an additional 22 h. A CCK-8 assay was then performed to measure cell viability. The absorption values were measured at 540 nm using a microplate reader.

### Nitric Oxide (NO) Assay

RAW264.7 cells were incubated with different concentrations of obacunone (0–100 μM) for 2 h before being stimulated by LPS (1 μg/mL) for an additional 22 h. The supernatant was then collected and a Griess assay was performed to measure the relative NO secretion levels in each group. Finally, the absorbance of each well was measured at 450 nm using a microplate reader as previously described (Luo et al., 2017).

### Immunoblotting and RNA Analysis

Colon segments (∼1.5 cm near the anus) or cultured cells were homogenized or lysed in lysis buffer (Thermo Fisher Scientific, Mannheim, MA, United States) containing protease and phosphatase inhibitor cocktail tablets (Roche Diagnostics GmbH, Mannheim, GER). The lysate was centrifuged (4°C, 12,000 × *g*, 15 min) and the supernatant was collected. The procedure for immunoblotting was performed as previously described (Yue et al., 2018). In brief, proteins (30 μg) was separated by 10% SDS-PAGE and transferred onto a PVDF membrane. The membrane was blocked in 5% (w/v) skim milk for 2 h at room temperature and immunoblotted with primary antibody. Then blots were washed and incubated with HRP-coupled secondary antibody at room temperature. Finally, the blots were observed by enhanced chemiluminescence (ECL) detection reagents. Protein expressions were analyzed by a GS-700 imaging densitometer (Bio-Rad, CA, United States). β-actin (Santa Cruz, CA, United States) was used as an internal control.

RAW264.7 cells were treated with different concentrations of obacunone (0–100 μM) for 2 h before being stimulated by LPS (1 μg/mL) for an additional 22 h. Total RNA was extracted from cultured cells using TRIzol reagent. Reverse transcription and qPCR were carried out using SYBR Premix ExTaq Mix in an ABI Prism 7900HT Sequence Detection System (Life Technologies, Carlsbad, CA, United States) as previously described (Dou et al., 2012). The following thermal cycler parameters were used: 1 cycle of 95°C for 30 s and 40 cycles of denaturation (95°C, 5 s) and combined annealing/extension (60°C, 30 s). Relative mRNA expression levels were calculated by the comparative Ct method, and the values were normalized as the ratio of the optimal density relative to β-actin. The sequences of the primers were listed in Table 1.

**TABLE 1:** The list of primers used for qPCR.

Gene	Primer sequence (5′–3′)
m IL-1α	F: ATGACCTGCAACAGGAAGTAAAA
	R:TGTGATGAGTTTTGGTGTTTCTG
m IL-1β	F: ATTGTGGCTGTGGAGAAG
	R: AAGATGAAGGAAAAGAAGGTG
m IL-16	F: GATACCACAGCCGAAGACCCTTGG
	R: GTGCTCGCTGGCTAGGCATCTTG
m iNOS	F: ATTGTGGCTGTGGAGAAG
	R: AAGATGAAGGAAAAGAAGGTG
m COX-2	F: GCCTTCCCTACTTCACAA
	R: ACAACTCTTTTCTCATTTCCAC
m β-actin	F: GGGAAATCGTGCGTGAC
	R: AGGCTGGAAAAGAGCCT

### Microbiota Sequencing Analysis

Mice feces were collected and stored at −80°C. Genomic DNA was extracted from the fecal samples using the E.Z.N.A. Soil DNA Kit (Omega Bio-tek, GA, United States), according to the manufacturer’s protocol. A NanoDrop 2000 UV-vis spectrophotometer (Thermo Fisher Scientific, DE, United States) was used to assess the concentration and quality of the DNA. The V3–V4 hypervariable regions of the bacterial 16S rRNA gene were amplified using primers 338F (5′-ACTCCTACGGGAGGCAGCAG-3′) and 806R (5′-GGACTACHVGGGTWTCTAAT-3′) in a GeneAmp 9700 ABI thermocycler PCR system (Carlsbad, CA, United States). PCR reaction was conducted using the following cycling conditions: an initial denaturation at 95°C for 3 min, followed by 27 cycles of denaturation at 95°C for 30 s, annealing at 55°C for 30 s, and elongation at 72°C for 45 s, and a final extension at 72°C for 10 min. Sequencing was performed using the high-throughput Illumina MiSeq platform (Illumina, CA, United States) according to standard protocols (Majorbio, Shanghai, China). Raw fastq files were quality-filtered by Trimmomatic and merged by FLASH. All the results were based on sequenced reads and operational taxonomic unit (OTU) clustering was performed with a 97% similarity cutoff and a 70% confidence threshold.

### Statistics

Significance between groups was evaluated by one-way analysis of variance (ANOVA) using GraphPad Prism 7 software (GraphPad Software, La Jolla, CA, United States). All data were presented as the mean ± standard deviation (SD). *p*-values < 0.05 (two-sided) were considered significant (^∗^*p* < 0.05, ^∗∗^*p* < 0.01, ^∗∗∗^*p* < 0.001). All the 16S rDNA sequencing data were analyzed on the online Majorbio I-Sanger Cloud Platform^1^.

## Results

### Obacunone Exerted Protective Effects Against DSS-Induced Colitis in Mice

**FIGURE 1:**
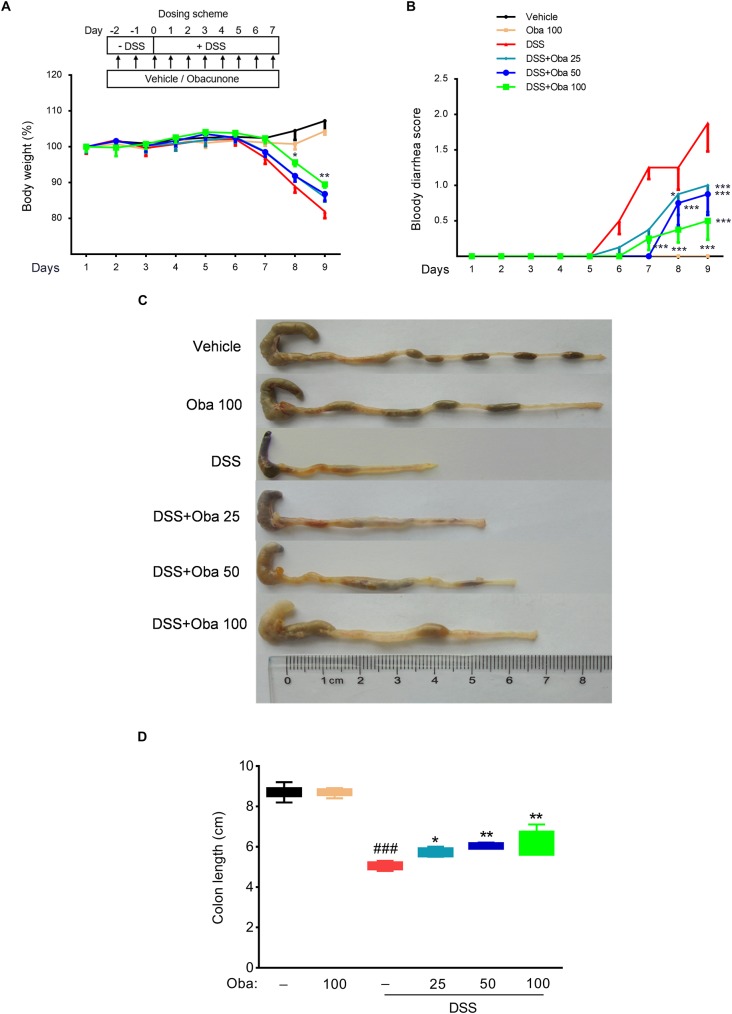
Obacunone ameliorated body weight loss, bloody diarrhea, and colon shortening in IBD model mice. **(A)** Body weight was recorded after DSS induction of colitis. Data were plotted as a percentage of basal body weight. **(B)** The occurrence of bloody diarrhea. Data were plotted as the percentage of the total number of mice that had bloody diarrhea at different time points of DSS treatment. Macroscopic observation **(C)** and assessment of colon shortening **(D)** at the end of the study. Data were expressed as the mean ± SD (*n* = 6 mice per group). *p < 0.05, **p < 0.01, ***p < 0.001 vs. the DSS-treated group; ^###^*p* < 0.001 vs. the control group.

Dextran sulfate sodium-induced colitic mice exhibited body weight loss, accompanied by diarrhea, and bloody stools, compared with vehicle- or obacunone only-treated mice. However, treatment with obacunone (50 or 100 mg/kg) significantly attenuated disease symptoms in DSS-treated mice, including weight loss, diarrhea, bloody stool, and colonic shortening (Figures 1A–D). Moreover, the mice in the obacunone (100 mg/kg)-only treatment group showed an almost identical disease hallmark to that of vehicle-treated mice, indicating that obacunone had no obvious toxic side effects.

**FIGURE 2:**
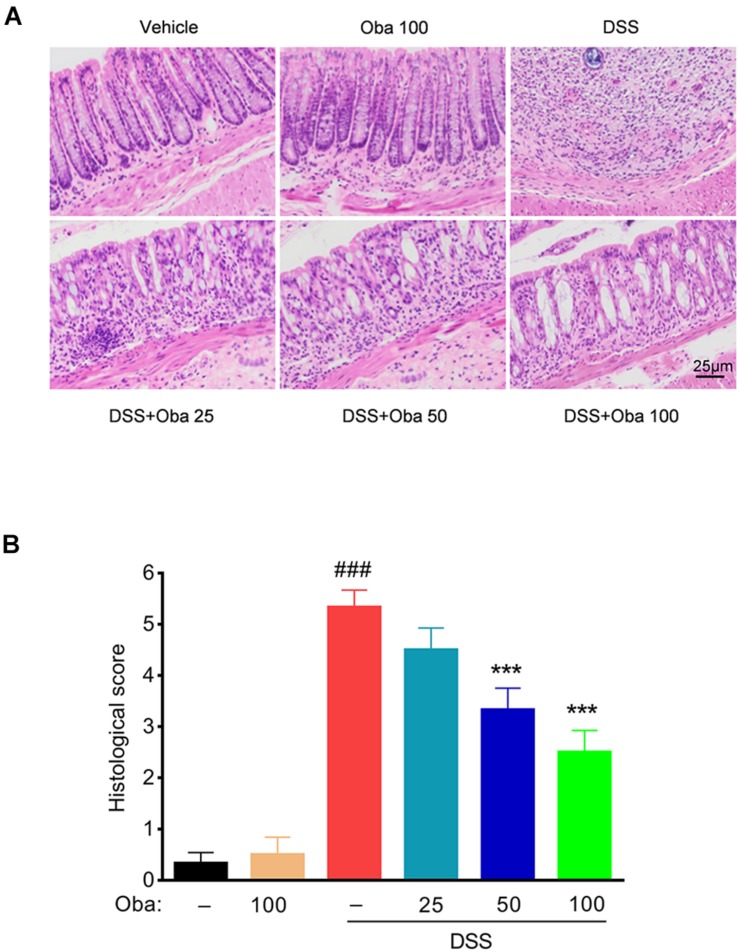
Obacunone ameliorated inflammatory infiltration and histopathological injury in IBD model mice. Representative hematoxylin–eosin (H&E)-stained colon sections **(A)** and histological score **(B)**. Scale bar, 25 μm. Data were expressed as the mean ± SD (*n* = 6 mice per group). ***p < 0.001 vs. the DSS-treated group; ^###^*p* < 0.001 vs. the control group.

Additionally, examination of pathological colon tissue sections showed that DSS treatment resulted in severe intestinal epithelial injury, including crypt loss, mucosal ulceration, muscle thickening, and neutrophil infiltration. Obacunone (25, 50, or 100 mg/kg)-treated mice presented reduced loss of mucosal architecture, fewer ulcerations, and less cellular infiltration (Figures 2A,B). Because obacunone treatment at 100 mg/kg produced the best phenotypes, 100 mg/kg treatment group was used in the subsequent experimental analyses.

### Obacunone Exerted a Modulating Effect on the Disordered Gut Microbiota of IBD Mice

**FIGURE 3:**
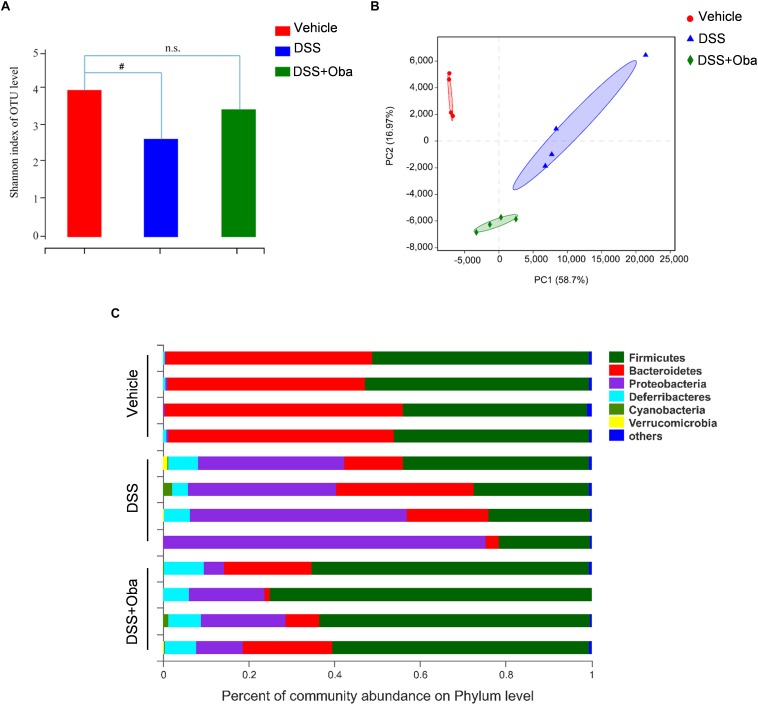
Obacunone modulated the composition of gut microbiota in DSS-treated mice. **(A)** Index-group Difference Test of Shannon Value in Sample Hierarchical Cluster Tree alpha-Diversity. Student’s *t*-test. Classification level: OTU. **(B)** Graph of principal component analysis (PCA) at the genus level. **(C)** The proportion of dominant communities at the phylum level in each group of samples. Phylum-level communities presenting in less than 5% of the samples were merged into others. Data = mean (*n* = 4 mice per group). ^#^*p* < 0.05 vs. the control group; n.s., no significance.

**FIGURE 4:**
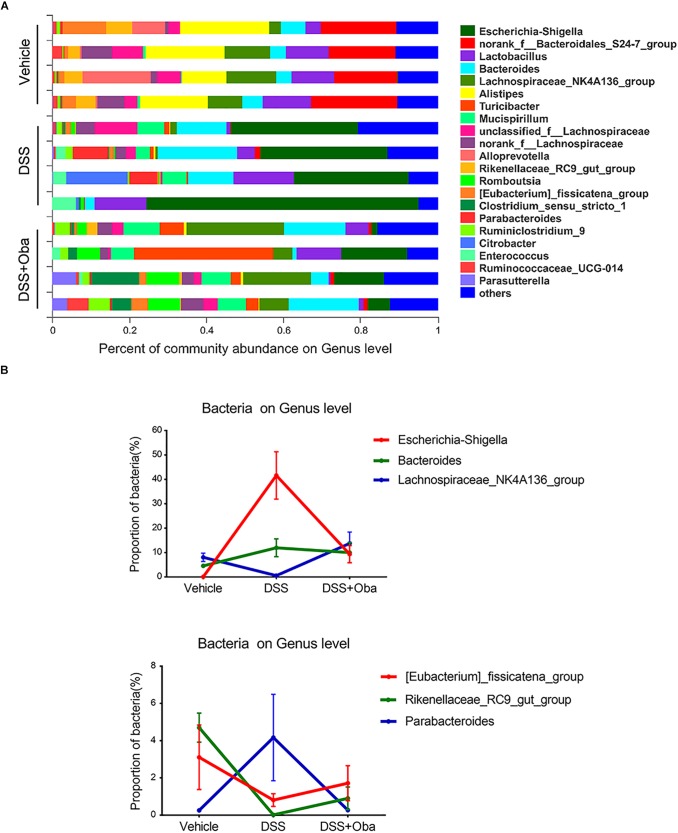
Composition and abundance of gut microbiota at the genus level. **(A)** The proportion of dominant genera in each group of samples. Genus-level communities presenting in less than 5% of the samples were merged into others. **(B)** Distribution of *Escherichia*–*Shigella*, *Bacteroides*, Lachnospiraceae_NK4A136_group, *(Eubacterium)_fissicatena_group*, *Rikenellaceae_RC9_gut_group*, and *Parabacteroides* in each group. Data were expressed as the mean ± SD (*n* = 4 mice per group).

Bacterial 16S rRNA gene sequencing was used to evaluate the effect of obacunone on DSS-induced changes in gut microbiota composition. Principal component analysis (PCA) revealed that each group was clustered separately (Figures 3A,B). The Shannon index was used to characterize the overall microbial diversity. The results showed that microbial diversity was significantly decreased in DSS-treated mice, whereas obacunone treatment mitigated these changes (Figure 3A), although the difference was not significant. The major intestinal bacteria at the phylum level included *Firmicutes*, *Bacteroidetes*, *Proteobacteria*, *Deferribacteres*, *Cyanobacteria*, and *Verrucomicrobia* (Figure 3C). Moreover, DSS treatment decreased the relative abundance of *Bacteroidetes* and enriched the abundance of *Proteobacteria* compared with that of the vehicle-treated group; however, obacunone treatment mitigated the DSS-induced phylum-level changes Supplementary Table 1. At the genus level, the DSS-treated group exhibited proportional decreases in the abundances of *Lachnospiraceae-NK4A136-group*, *Rikenellaceae-RC9-gut-group*, and *(Eubacterium)-fissicatena-group*, but these changes were attenuated by obacunone administration (Figures 4A,B). Furthermore, the abundance of some pathogenic bacteria such as *Escherichia–Shigella* and *Enterococcus*, was significantly increased in DSS-treated group, whereas the abundance of *Escherichia–Shigella* and *Enterococcus* was decreased as a result of obacunone treatment (Figures 4A,B and Supplementary Table 2). Collectively, our results revealed that DSS treatment disturbed gut microbiota homeostasis, and this gut microbiota imbalance could be reversed by obacunone treatment.

### Obacunone Exerted a Protective Effect on the Integrity of the Intestinal Barrier in Colitic Mice

**FIGURE 5:**
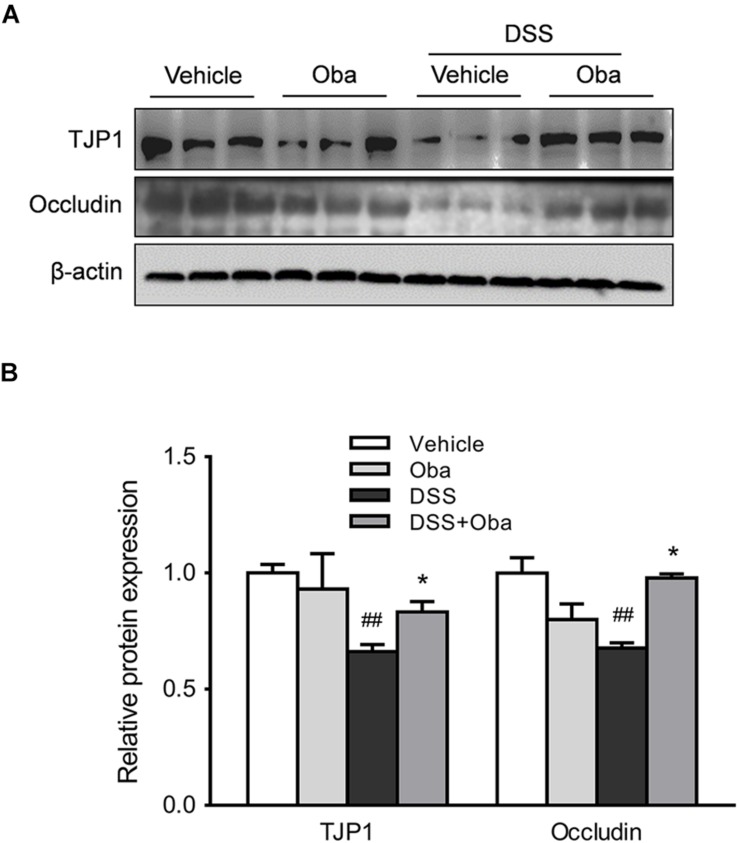
Obacunone inhibited the DSS-induced loss of TJP1 and occludin protein expression. Effect of obacunone on tight junction protein (TJP1 and occludin) expression in colonic tissues. Representative western blots **(A)** and quantitative analysis **(B)** of the TJP1 and occludin proteins. Data were expressed as the mean ± SD (*n* = 3 mice per group). *p < 0.05 vs. the DSS-treated group; ^##^*p* < 0.01 vs. the control group.

To assess the integrity of the intestinal barrier in colitic mice, we determined the expression levels of the tight junction proteins, TJP1 and occludin, in colonic tissue of DSS-induced colitic mice. The results showed that the expression levels of TJP1 and occludin were markedly downregulated in DSS-treated mice (Figures 5A,B). However, obacunone treatment significantly rescued the reduced expression of these barrier proteins, indicating that obacunone might exert a protective effect against DSS-induced disruption of intestinal epithelial barrier integrity.

### Obacunone Inhibited the TLR4/NF-κB Signaling Cascade in Colitic Mice

**FIGURE 6:**
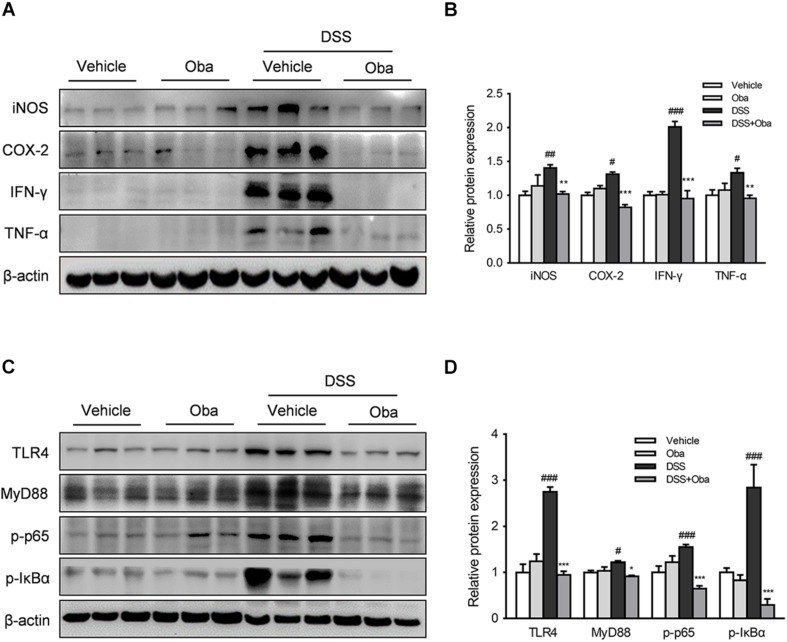
Obacunone inhibited TLR4/NF-κB signaling *in vivo*. After the mice had been euthanized, colon samples were removed and evaluated. **(A,B)** Effect of obacunone on the protein expression levels of inflammatory mediators in colon tissue. Western blot analysis of the expression of iNOS, COX-2, IFN-γ, and TNF-α in each group. A representative blot is shown. The expression levels of all the proteins were normalized to actin. **(C,D)** Western blot analysis of the expression of TLR4, MyD88, p-IκBα, and p-p65 in each group. A representative blot is shown. Data were presented as the mean ± SD (*n* = 3). *p < 0.05, **p < 0.01, ***p < 0.001 vs. the DSS-treated group; ^#^*p* < 0.05, ^##^*p* < 0.01, ^###^*p* < 0.001 vs. the control group.

To investigate the mechanisms underlying obacunone-associated attenuation in DSS-induced intestinal inflammation, we evaluated the effect of obacunone on the protein expression levels of inflammatory mediators. Significant increase in the expression levels of nitric oxide synthase, cyclooxygenase 2 (COX-2), interferon gamma (IFN-γ), and TNF-α was observed in the colon of DSS-induced colitic mice (Figures 6A,B). However, obacunone administration greatly reduced the expression levels of these inflammatory mediators in the colons of colitic mice. Moreover, the protein expression levels of TLR4, myeloid differentiation primary response gene 88 (MyD88), p-p65 (a subunit of NF-κB), and p-IκBα (an inhibitor of NF-κB) in colonic tissue was significantly increased after DSS treatment (Figures 6C,D). However, all these abnormally increased expression levels were markedly downregulated in colitic mice with obacunone treatment. Combined, these results indicated that obacunone exerted a protective effect against DSS-induced colitis through attenuation of TLR4/NF-κB signaling cascades.

### Obacunone Decreased the LPS-Induced Production of Proinflammatory Mediators in RAW264.7 Macrophages

**FIGURE 7:**
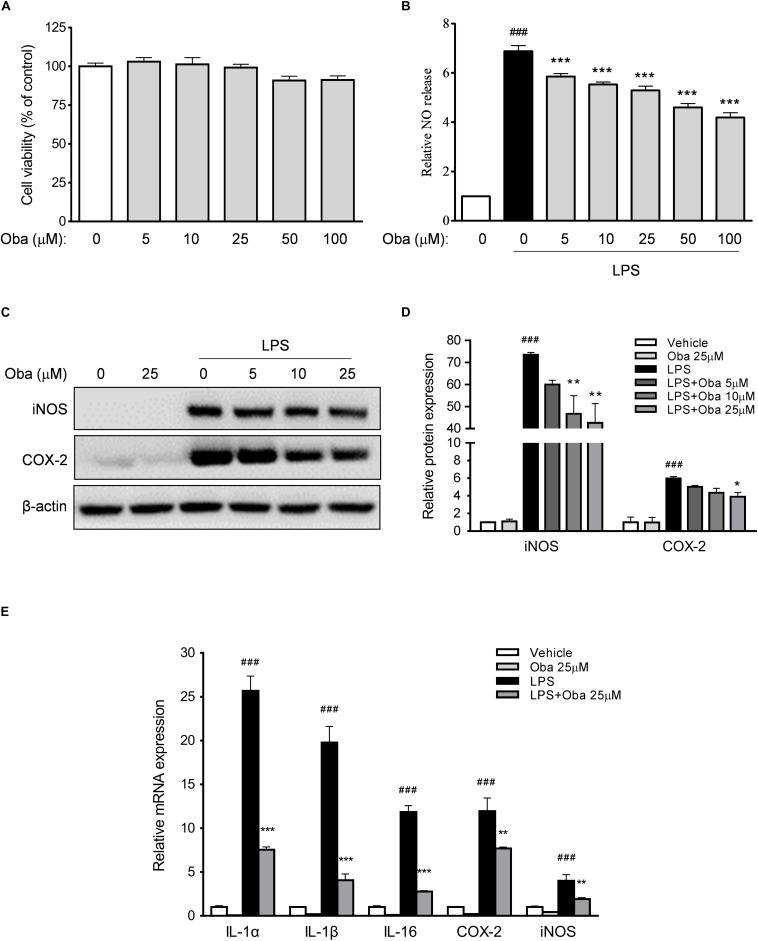
Obacunone inhibited the expression of pro-inflammatory mediators *in vitro*. **(A,B)** Effect of obacunone on the viability and NO secretion levels of LPS-induced RAW264.7 macrophages. Cells were treated with obacunone (0–100 μM) for 24 h, after which cell viability and NO production were determined. **(C,D)** RAW264.7 macrophages were treated as described in section “Materials and Methods.” The protein expression levels of iNOS and COX-2 in RAW264.7 cells were determined by western blot. Protein expression levels were normalized to that of actin. **(E)** Effect of obacunone on the mRNA expression of proinflammatory mediators in LPS-induced cells. RAW264.7 macrophages were treated as described in section “Materials and Methods,” following which the mRNA expression levels of IL-1α, IL-1β, IL-16, COX-2, and iNOS were determined by qRT-PCR. All mRNA expression levels were normalized to that of actin. Data were presented as the mean ± SD (*n* = 3). *p < 0.05, **p < 0.01, ***p < 0.001 vs. the DSS-treated group; ^###^*p* < 0.001 vs. the control group.

RAW264.7 mouse macrophages were used to further evaluate the anti-inflammatory effects of obacunone. The initial cytotoxicity evaluation suggested that obacunone was almost non-cytotoxic at dosages up to 100 μM (Figure 7A). Additionally, we found that obacunone markedly suppressed LPS-stimulated NO production in RAW264.7 cells in a concentration-dependent manner (0–100 μM) (Figure 7B). Meanwhile, the protein expression levels of the proinflammatory mediators iNOS and COX-2 were significantly increased in RAW264.7 cells after exposure to LPS. However, obacunone concentration-dependently decreased the LPS-induced increase in the levels of iNOS and COX-2 (Figures 7C,D). We then quantified the mRNA expression levels of pro-inflammatory mediators. The results showed that the mRNA levels of IL-1α, IL-1β, IL-16, COX-2, and iNOS were markedly upregulated in LPS-stimulated macrophages; however, obacunone treatment attenuated the LPS-induced increase in the mRNA levels of these proinflammatory mediators (Figure 7E).

### Obacunone Exerted a Protective Effect on Intestinal Epithelial Barrier Integrity in Epithelial Cells

**FIGURE 8:**
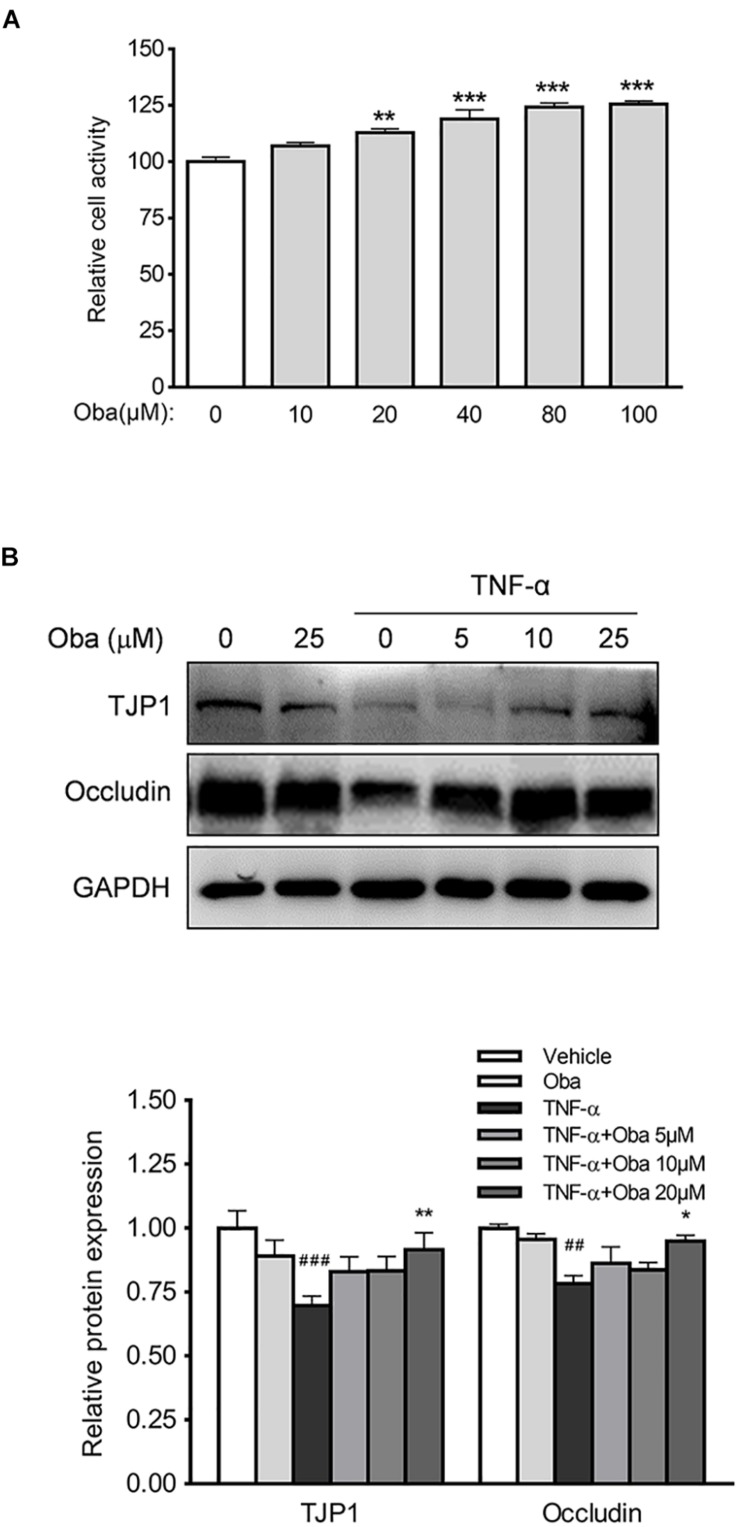
Obacunone inhibited TNF-α-induced loss of TJP1 and occludin. **(A)** Effect of obacunone on the viability of NCM460 cells. The cells were treated with obacunone (0–100 μM) for 24 h and cell viability was then measured. **(B)** Effect of obacunone on the expression of the tight junction proteins TJP1 and occludin in TNF-α-stimulated NCM460 cells. Representative western blots and quantitative analysis of TJP1 and occludin protein. Data were expressed as the mean ± SD (*n* = 3 mice per group). *p < 0.05, **p < 0.01, ***p < 0.001 vs. the DSS-treated group; ^##^*p* < 0.01, ^###^*p* < 0.001 vs. control group.

The human colonic epithelial cell line NCM460 is commonly used to investigate the mechanisms of inflammatory damage and repair in intestinal epithelial barrier (Tsoi et al., 2017). We found that obacunone markedly increased NCM460 cell viability in a concentration-dependent manner (Figure 8A). Further, our study showed that TNF-α treatment downregulated the expression levels of the tight junction proteins TJP1 and occludin; however, these decreased expression levels were significantly reversed by obacunone treatment (Figure 8B).

## Discussion

Ulcerative colitis is a refractory inflammatory condition that affects the digestive tract, and the lesions are mainly located in the colon, rectum, or entire colorectal mucosa. Although the global incidence of UC has shown a gradual increase (Yue et al., 2018), therapeutic drugs with limited side effects are still not available for the treatment of UC patients. Consequently, the pathogenesis of UC and the optimal treatment approaches for this condition have attracted increasing attention in recent years (Zhang and Li, 2014).

Animal models with clinical characteristics and pathological changes similar to those observed in UC patients are important tools for investigating drug discovery, action mechanisms, and therapeutic exploration associated with the pathogenesis of UC (Bamias et al., 2017). As a well-known experimental UC model, the DSS-induced disease symptoms of disrupted epithelial barrier function and thereby increased exposure of innate immune elements in lamina propria to the invasive gut microbiota, are distinguished and are resemble with that of UC patients (Eichele and Kharbanda, 2017). In the current study, we found that mice with DSS treatment exhibited severe intestinal inflammation, which was accompanied with body weight loss, bloody diarrhea, the formation of large ulcers, diffuse necrosis, and neutrophil infiltration. However, obacunone treatment markedly alleviated these DSS-induced symptoms of colitis. Moreover, no toxic reaction was observed in the obacunone-treated mice, suggesting the relative safety for obacunone’s potential clinical application.

Changes in the composition of gut microbiota were recently shown to be highly correlated with the incidence of UC. Severe dysbacteriosis has been reported in patients with UC, which is reflected in a reduced abundance of the commensal intestinal bacteria, *Firmicutes* and *Bacteroidetes*, and an increased abundance of pathogenic *Proteobacteria* (Ni et al., 2017; Sommer et al., 2017). Through 16S rRNA gene sequencing, we also found differences in gut bacterial community composition between DSS-treated group and normal control group, which included a decrease in the commensal intestinal bacteria (such as *Firmicutes* and *Bacteroidetes*) and an increase in pathogenic bacteria (such as *Proteobacteria*). However, after obacunone treatment, there was a shift in the composition of the bacterial community, which became more alike that of the vehicle group, indicative of an improving trend. Furthermore, a new study showed that there is a greater abundance of *Escherichia*–*Shigella* (a *Proteobacteria*) in the inflamed mucosae than in non-inflamed mucosae in UC patients (Baumgart et al., 2007; Xu et al., 2018). Moreover, *Escherichia*–*Shigella* is a Gram-negative bacterium that invades the human colonic epithelium and induces inflammatory responses owing to the presence of LPS in its outer membrane (Gronbach et al., 2014; Stephens and von der Weid, 2019). In our study, DSS treatment also led to an increase in the abundance of *Escherichia*–*Shigella*; however, this increase was attenuated with obacunone treatment.

Toll-like receptor 4, as the first identified human toll-like receptor, has been extensively investigated. TLR4 recognizes the exogenous bacterial ligand, LPS, and activates a signaling cascade that leads to pro-inflammatory response to destroy the invading pathogens (Liu et al., 2014; Holdbrook et al., 2019). Activation of TLR4 is known to trigger both MyD88-dependent and MyD88-independent pathways, which in turn lead to the activation of NF-κB and production of inflammatory mediators (O’Neill, 2002). It is widely accepted that TLR4 is a double-edged sword, as minor activation of TLR4 is essential in the maintenance of immune homeostasis, while excessive TLR4 activation can lead to the induction of host inflammatory responses (Steimle et al., 2019). TLR4 is highly expressed in the colon tissues of UC patients and DSS-induced colitic mice, and is considered to be a contributing factor to the initiation and development of intestinal inflammation in UC (Taniguchi et al., 2009; Murad, 2014; Yue et al., 2019). We found that the protein expression levels of TLR4, MyD88, p-p65, and p-IκBα were significantly increased in DSS-induced colitic mice; however, the expression levels of TLR4 and its downstream proteins were markedly decreased by obacunone treatment. Our results were consistent with the previous reports (Shi et al., 2019).

Furthermore, as the first line of immune defense, the intestinal epithelial barrier is crucial for the protection of the host against invasive pathogenic bacteria or viruses. With the loss of intestinal barrier integrity, bacteria (e.g., *Escherichia*–*Shigella*) and toxic substances (e.g., LPS) can penetrate through the intestinal wall and trigger the aforementioned feedback cycle (Chelakkot et al., 2018; Marin et al., 2019). TJPs are vital for the maintenance of epithelium integrity, and dysregulation of tight junctions is frequently correlated with the loss of intestinal barrier integrity (Turner et al., 2014). In our study, we used a human intestinal epithelial cell line NCM460 to explore the effects of obacunone on the expression of TJPs. Interestingly, we found that the protein expression levels of TJP1 and occludin were increased by obacunone treatment, whereas the levels of the barrier proteins were decreased in UC mice, as well as in TNF-α-stimulated NCM460 cells.

## Conclusion

**FIGURE 9:**
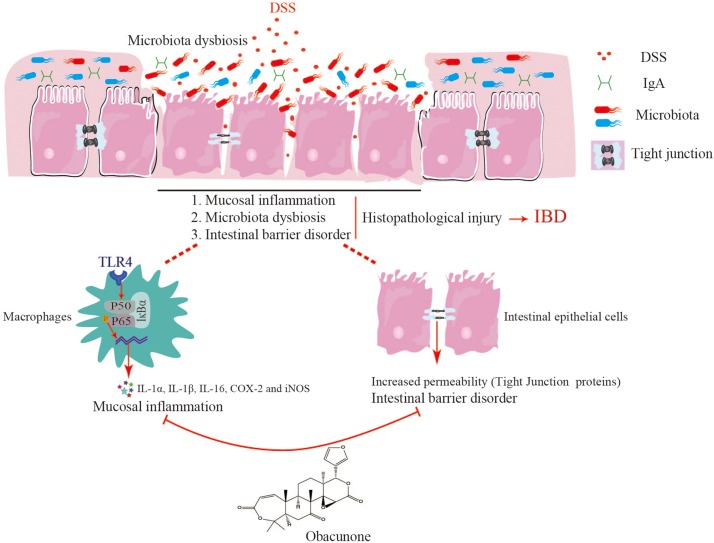
Obacunone presented an anti-colitis efficacy, at least partially, via the modulation of the gut microbiota and attenuation of TLR4/NF-κB signaling cascades, as well as protection against disruption of the intestinal epithelial barrier.

In summary, for the first time, our study indicated that obacunone alleviated intestinal damage, bloody stools, and diarrhea symptoms in DSS-induced colitic mice. Moreover, the anti-inflammatory effect of obacunone was exerted, at least partially, via the modulation of the gut microbiota and attenuation of TLR4/NF-κB signaling cascades, as well as protection against disruption of the intestinal epithelial barrier (Figure 9). Importantly, non of mice that received obacunone alone exhibited apparent clinical lesions or mucosal damage throughout the study, indicating the relative safety of obacunone management. The current results strongly suggest that obacunone could be a potential therapeutic drug in colitis. This work allow us to hypothesized the potential application of obacunone on other bowel diseases such as Crohn’s disease and colitis associated colorectal cancer. However, the further studies are required to confirm these hypotheses.

## Data Availability Statement

The raw data supporting the conclusions of this article will be made available by the authors, without undue reservation, to any qualified researcher.

## Ethics Statement

The animal study was reviewed and approved by the Animal Experimentation Ethics Committee at Shanghai University of Traditional Chinese Medicine (Animal license key: SYXK2014-0008).

## Author Contributions

ZW and WD conceived and designed the experiments. XL, BY, ZY, YR, JZ, and JR performed the experiments and collected the data. XL, BY, ZY, and YR analyzed the data. BY and WD wrote the manuscript. XL, ZW, and WD supervised the study.

## Conflict of Interest

The authors declare that the research was conducted in the absence of any commercial or financial relationships that could be construed as a potential conflict of interest.

## Footnotes

1 www.i-sanger.com
