# Supplementary material for: Obacunone Protects Against Ulcerative Colitis in Mice by Modulating Gut Microbiota, Attenuating TLR4/NF-κB Signaling Cascades, and Improving Disrupted Epithelial Barriers
Source: Front Microbiol. 2020 Mar 31;11:497. doi: 10.3389/fmicb.2020.00497 (PMC7136403; doi:10.3389/fmicb.2020.00497)
Supplement: Supplementary file 1 [file Table_1.docx]

Supplementary table 1. Relative abundance of the most representative Phylum

| Taxonomic  Phylum | Vehicle | DSS | DSS+Oba |
| --- | --- | --- | --- |
|  | Relative abundance (%) | | |
| *Firmicutes* | 47.88 ± 4.38 | 28.92 ± 10.01 ^##^ | 65.75 ± 6.41 ^***^ |
| *Bacteroidetes* | 50.67 ± 4.24 | 16.99 ± 12.06 # | 12.66 ± 9.67 |
| *Proteobacteria* | 0.33 ± 0.13 | 48.69 ± 19.41^###^ | 13.25 ± 6.83 ^**^ |
| *Deferribacteres* | 0.50 ± 0.35 | 4.25 ± 3.10 # | 7.60 ± 1.39 |
| *Cyanobacteria* | 0.015 ± 0.018 | 0.56 ± 1.00 | 0.47 ± 0.57 |
| *Verrucomicrobia* | 0.014 ± 0.017 | 0.32 ± 0.52 | 0.030 ± 0.054 |

***p* < 0.01, ****p* < 0.001 *vs.* the DSS-treated group; ^#^*p* < 0.05, ^#^*p* < 0.01, ^###^*p* < 0.001 *vs.* the control group.
