# Supplementary material for: Obacunone Protects Against Ulcerative Colitis in Mice by Modulating Gut Microbiota, Attenuating TLR4/NF-κB Signaling Cascades, and Improving Disrupted Epithelial Barriers
Source: Front Microbiol. 2020 Mar 31;11:497. doi: 10.3389/fmicb.2020.00497 (PMC7136403; doi:10.3389/fmicb.2020.00497)
Supplement: Supplementary file 2 [file Table_2.docx]

Supplementary table 2. Relative abundance of the most representative Genus

| Taxonomic  Genus | Vehicle | DSS | DSS+Oba |
| --- | --- | --- | --- |
|  | Relative abundance (%) | | |
| *Escherichia-Shigella* | 0.052 ± 0.055 | 41.63 ± 19.41 ^##^ | 9.35 ± 7.04 ** |
| *Lactobacillus* | 13.72 ± 4.42 | 8.67 ± 6.70 | 4.73 ± 5.13 |
| *Bacteroides* | 3.62 ± 1.86 | 11.99 ± 7.37 | 10.03 ± 8.31 |
| *Lachnospiraceae-*  *NK4A136-group* | 10.54 ± 2.18 | 0.56 ± 0.76 | 13.79 ± 9.37 |
| *f-Bacteroidales*  *-S24-7-group* | 22.62 ± 8.22 | 0.41 ± 0.64 ^###^ | 0.71 ± 0.53 |
| *Alistipes* | 14.71 ± 5.14 | 0.14 ± 0.22 ^###^ | 0.41 ± 0.32 |
| *Turicibacter* | 0.021 ± 0.030 | 0.74 ± 0.57 | 12.05 ± 16.13 |
| *Mucispirillum* | 0.50 ± 0.35 | 4.25 ± 3.01 ^#^ | 7.60 ± 1.39 |
| *f-Lachnospiraceae* | 5.102 ± 2.79 | 2.05 ± 2.26 | 3.62 ± 1.66 |
| *Romboutsia* | 0.0067 ± 0.0082 | 0.66 ± 0.42 | 6.38 ± 2.77 |
| *Clostridium-sensu*  *-stricto-1* | 0.11 ± 0.060 | 0.34 ± 0.20 | 5.19 ± 5.21 |
| *Parabacteroides*  *Citrobacter* | 0.17 ± 0.11 | 4.17 ± 4.65 | 0.27 ± 0.16 |
| *Citrobacter* | 0.00081 ± 0.016 | 4.18 ± 7.88 | 0.0090 ± 0.018 |
| *Alloprevotella* | 4.97 ± 8.62 | 0 ± 0 | 0 ± 0 |
| *Ruminiclostridium-9* | 0.55 ± 0.12 | 0.88 ± 0.88 | 3.33 ± 1.66 |
| *Rikenellaceae-RC9*  *-gut-group* | 3.70 ± 1.41 | 0.020 ± 0.025 ^##^ | 0.90 ± 1.22 |
| *Enterococcus* | 0 ± 0 | 3.15 ± 2.56 ^#^ | 0.75 ± 0.89 |
| *Ruminococcaceae*  *-UCG-014* | 1.58 ± 0.85 | 0.29 ± 0.35 | 1.80 ± 2.58 |
| *Parasutterella* | 0.097 ± 0.086 | 0.20 ± 0.18 | 2.50 ± 3.02 |

***p* < 0.01 *vs.* the DSS-treated group; ^#^*p* < 0.05, ^#^*p* < 0.01, ^###^*p* < 0.001 *vs.* the control group.
